# Molecular Studies and Advanced Visualization of the Trapping of Methane Nanobubbles during Hydrate Growth

**DOI:** 10.1021/acs.jpcb.4c07851

**Published:** 2025-04-07

**Authors:** Temitayo Adeyemi, Olufemi Olorode

**Affiliations:** Department of Petroleum Engineering, Louisiana State University, Baton Rouge, Louisiana 70803, United States

## Abstract

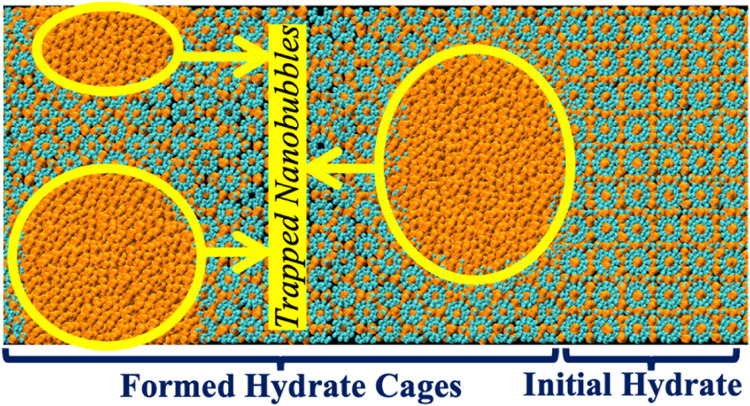

The potential application of gas hydrates in storing clean energy has increased the interest in studying clathrate hydrates of gases like methane, CO_2_, and hydrogen. In this work, we conduct large-scale molecular studies of methane hydrate growth and visualize the simulation results using mixed reality (MR) headsets and regular two-dimensional snapshots of the simulation domain. The results show the novel molecular observation of the trapping of gas nanobubbles within the growing solid hydrate. Our first-of-a-kind visualization of the internal hydrate structures in mixed reality enabled the length measurements of the simulation domain and nanobubble sizes, which showed that the gas nanobubbles were up to 9 nm in diameter. This is bigger than the simulation domain commonly used in atomistic gas hydrate studies, which explains why this is the first observation of the trapping of methane gas nanobubbles within a growing hydrate. Furthermore, our estimates of the increased storage due to the trapping of the nanobubbles indicate a 37% increase in the weight percentage of methane stored. Although this work focused on nanobubble-enhanced methane storage in hydrates, the idea, methods, and tools developed can be leveraged to enhance the storage of other gases, like hydrogen and CO_2_. This study also revealed that the presence of gas nanobubbles accelerates the rate of hydrate formation, which is consistent with experimental observations. Finally, we expect our workflow for MR visualization of gas hydrate structures to facilitate other novel observations and insights from molecular dynamics (MD) studies of gas hydrates.

## Introduction

Methane hydrates are ice-like crystalline compounds with methane gas molecules trapped within polyhedral cages formed by hydrogen bonds between water molecules.^1^ They typically require low temperature and high pressure to form.^2^ They are widely distributed in the continental margin, continental slope, and permafrost.^3^ At higher temperatures and/or lower pressures, methane hydrates melt or dissociate to release methane gas, a significant energy source today. Although methane is a fossil fuel, it is much cleaner than oil and coal because it releases less carbon dioxide per unit amount of energy.^4,5^ Naturally occurring methane hydrate deposits, also loosely referred to as natural gas hydrates, contain approximately twice as much as the total energy from all other fossil fuels combined.^6,7^ They also pose a severe threat to the petroleum industry because they can plug pipelines, resulting in significant financial losses and safety hazards.^8^

Although this work focuses on methane hydrates, it is worth noting that other gases can be trapped within gas hydrate cages. Of these different gas hydrates, CO_2_ and hydrogen hydrates have received considerable research interest over the past decade because of the increasing concerns about global warming. For instance, a few researchers have suggested hydrate-based CO_2_ capture,^9^ transportation,^10,11^ and storage in the subsurface^12^ or oceans.^13−16^ A few researchers have also suggested the idea of curtailing CO_2_ gas leakage from subsurface rocks to the surface by forming CO_2_ gas hydrates within gas hydrate stability zones (GHSZ).^17,18^ Additionally, some researchers^19−21^ have studied hydrate-based hydrogen storage (HBHS) as a potential clean energy technology because the combustion of pure hydrogen hydrates yields only water, which is environmentally friendly. Hydrogen hydrates have a unique potential as “hydrogen batteries”, which can be charged (during hydrate formation) or discharged (during dissociation) depending on the pressure and temperature imposed.^22^ Another potential use of gas hydrates is in water desalination^23^

It is essential to study the thermodynamics and kinetics of gas hydrate formation and dissociation to harness the potential of these hydrates. Several researchers^17,24^ have performed experiments to study the growth of gas hydrates in a pressure cell, whereas others^25,26^ have performed molecular dynamics (MD) studies to obtain molecular-level insights into the formation of gas hydrates. Most MD studies use atomistic force fields like the TIP4P-Ice model^27^ for water, which are generally considered to be more accurate than coarse-grained force fields like the monatomic water (mW) model.^28^ However, gas hydrate simulations performed using coarse-grained models can be up to 180 times faster than their atomistic counterparts. So, when carefully calibrated, like in the methane hydrate studies by Jacobson and Molinero,^29^ they provide a fast and accurate alternative to atomistic models. Using the calibrated Stillinger–Weber^30^ potential presented in Jacobson and Molinero,^29^ Adibifard and Olorode^31^ simulated methane hydrate dissociation in systems up to 100 times larger than previous studies. The results showed the formation of gas nanobubbles within the dissociating solid methane hydrate. The size of the nanobubbles was bigger than the simulation domain used in most previous studies, which explains why this phenomenon had not been observed before.

Previous MD studies of gas hydrates obtained insights into the formation and dissociation of gas hydrates by taking two-dimensional (2D) snapshots of the simulation domain’s sides/faces and cross sections. However, some of the limitations of this standard approach are as follows:

1. The entire hydrate structure typically moves across the periodic boundary condition on all the sides/faces of the simulation box, making it challenging to track features observed in the domain’s interior by simply taking multiple cross sections. For instance, tracking the nucleation and growth of gas nanobubbles in Figure 4 of Adibifard and Olorode^31^ was difficult because the gas nanobubbles moved from one slice to the other during the simulation.
2. All gas hydrate cages are not necessarily parallel to the faces of the simulation box during the simulation, making it hard or impossible to observe everything occurring within all the cages in the simulation domain from 2D snapshots of its faces or cross sections.
3. Taking and visualizing several snapshots of the cross sections of the simulation domain in the *x*, *y*, and *z* directions and over several time steps is tedious. This implicitly curtails the timely observation of new insights that could be gleaned from MD simulation results.

This work addresses all three limitations by leveraging cutting-edge advances in mixed reality (MR) and virtual reality (VR) visualization to digitally interact with the internal hydrate structures at different time steps, using Meta Quest VR headsets.^32^ Advances in MR and VR technologies can facilitate better molecular-level observation and understanding of physical phenomena like nucleation, phase transition, and diffusion.^33^ The idea of interactive molecular dynamics in virtual reality (iMD-VR) has enabled researchers to interact with molecular structures at the atomic scale.^34^ It has been applied to study enzyme catalysis,^35^ protein ligands coordination,^34^ movement of small molecules through solid materials like zeolites,^36^ and to visualize molecular geometry and wave function information in reactive MD simulations.^37^ VR has been applied for the real-time visualization and manipulation of the physical properties of systems like the HIV protease-cyclic urea inhibitor complex^38^ and to study various protein joints and other complex protein conformational changes.^39−42^ It has also been applied for the interactive simulation and VR visualization of guest molecules in a metal–organic framework (MOF). This has facilitated a better understanding of guest molecules adsorption in MOFs.^43^ Finally, VR software packages like Nanome, UnityMol, Peppy, and ProteinVR have facilitated our understanding of the dynamic interactions and bond formation between protein and drug candidates.^44−47^

Despite the various applications of VR in the different molecular studies discussed, the authors are unaware of any application of this technology to probe the internal structures of gas hydrates. The overarching objective of this study is to obtain new insights into methane hydrate growth by performing large-scale simulations and visualizing the results in MR. Although we typically use the terms VR and MR interchangeably, VR is restricted to the virtual environment. In contrast, MR allows us to interact with the virtual environment and physical objects in real life. We leveraged mixed reality in this work by using a physical measuring tape to measure the simulation box length and the gas nanobubbles’ diameters.

We used the coarse-grained intermolecular potential presented in Jacobson and Molinero^29^ to simulate methane hydrate growth in simulation boxes measuring 96.24, 12.03, and 12.03 nm in the *x*, *y*, and *z* directions. The rest of this paper starts with a summary of the coarse-grained force fields, initial hydrate configuration, an approach to estimate the hydrate growth rate, and a workflow to visualize the molecular trajectories in MR. Next, we discussed our observations of the trapping of gas nanobubbles within the growing solid hydrate. We presented an approach to quantify the degree to which the nanobubbles enhance hydrate-based natural gas storage. Finally, we showed the geometry of the gas nanobubbles using MR and concluded with the effects of these nanobubbles on hydrate growth rate.

## Materials and Methods

This section presents the coarse-grained potential used, the initial hydrate configuration, an approach to estimate the hydrate growth rate, and a workflow to visualize the hydrate internal structures in MR. The MD simulations were conducted using the Large-scale Atomic/Molecular Massively Parallel Simulator (LAMMPS).^48^

### Coarse-Grained Force Fields

We used the monatomic water (mW) model developed by Molinero and Moore^28^ to represent water interactions accurately and efficiently. It is based on the Stillinger–Weber (SW) potential,^29^ which essentially sums a pairwise/two-body interaction term (Φ_2_(*r*_*ij*_)) and a three-body interaction term (Φ_3_(*r*_*ij*_,*r*_*ik*_,θ_*ijk*_)), as follows
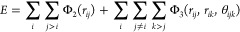
1where Φ_2_(*r*_*ij*_) is given as
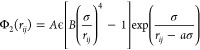
2and Φ_3_(*r*_*ij*_,*r*_*ik*_,θ_*ijk*_) is given as
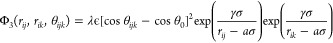
3The interaction term (λ) is essentially a weighting factor that controls how much Φ_3_ penalizes Φ_2_ to encourage the tetrahedral configuration of water. The symbols σ, ϵ, *r*_*ij*_, and θ_*ijk*_ represent the size scale, energy scale, distance between the *i*–th and *j*–th particles, and the angle between the *i*–*j* and *i*–*k* position vectors, respectively. The values of the parameters *A*, *B*, γ, *a*, and θ_0_ are given as 7.049556277, 0.6022245584, 1.2, 1.8, and 109.5°, respectively.^29^ Jacobson and Molinero^29^ provide the values of σ and ϵ for water–water, methane–methane, and water–methane interactions. Both water and methane molecules are modeled as particles in the coarse-grained simulations presented in this work.

### Standard Post-Processing of Simulation Results

We postprocessed the output LAMMPS trajectories using visual molecular dynamics (VMD).^49^ As in Adibifard and Olorode,^31^ we took several snapshots of the front and cross sections of the simulation domain to obtain insights into the processes occurring within the simulation box. Unfortunately, this approach is limited, as explained in the introduction. Our approach to address this limitation is discussed in the following subsection.

### Advanced Visualization with Mixed Reality (MR) Headsets

To obtain more conclusive insights into the processes occurring in the MD simulations conducted, we developed an MR visualization workflow that allows us to walk around, rotate, and zoom into the simulation domain in three dimensions (3D). The workflow leverages two open-source packages–ChimeraX^45^ and the LookSee Quest Molecular Viewer. We exported the “.gro” files from our LAMMPS trajectories in VMD. The “.gro” files were then visualized in ChimeraX and sent wirelessly from ChimeraX to the Meta Quest VR/MR headsets.

### Initial Hydrate Configuration

**Figure 1:**
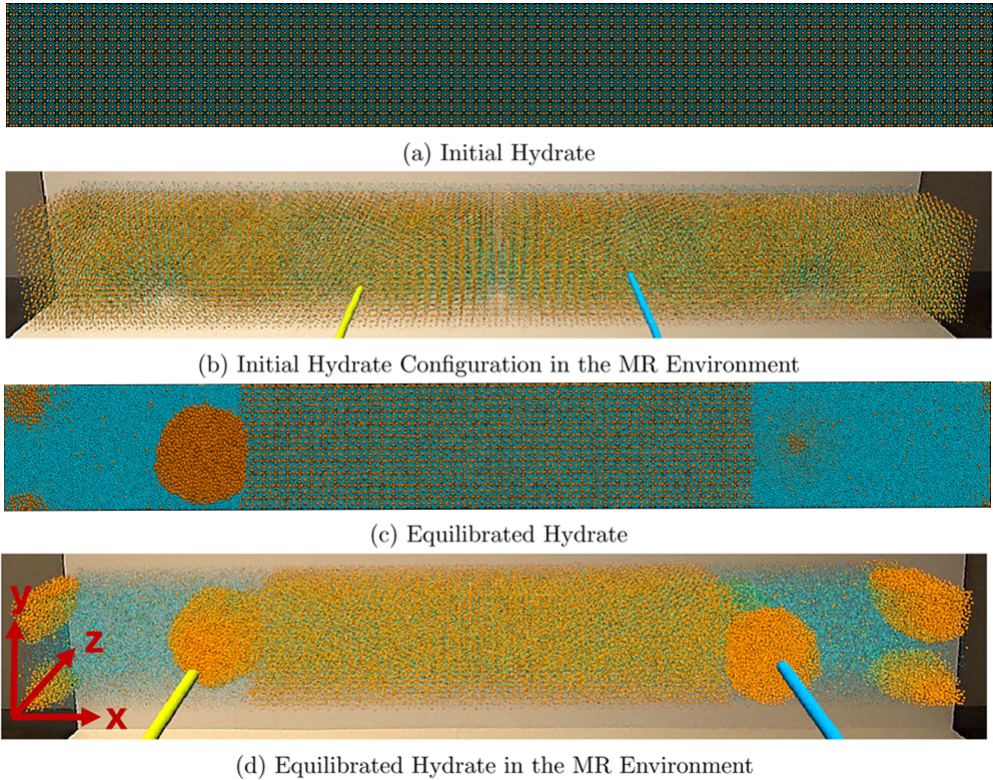
Images show (a) a regular 2D VMD screenshot of the initial hydrate at 250 K and 100 atm, (b) the corresponding MR snapshot of the initial hydrate, (c) a 2D VMD snapshot of the hydrate/fluid mixture equilibrated at 283 K and 100 atm, and (d) the corresponding MR snapshot of the equilibrated hydrate system. The orange and cyan spheres represent the methane and water molecules, respectively. The yellow and blue sticks are the VR handles (or controllers) for controlling the orientation and scale of the simulation box in the MR environment. The *X*, *Y*, and *Z* axes for all images are shown in panel (d).

We simulated a system with 80, 10, and 10 sI unit cells of methane hydrate in the *x*, *y*, and *z* directions, respectively. This yielded a simulation domain with a total of 432,000 molecules and an initial dimension of 96.24, 12.03, and 12.03 nm in the *x*, *y*, and *z*, respectively. We used periodic boundary conditions (PBC) on all sides or faces of the simulation domain. To equilibrate the hydrates, we performed a 500 ps simulation of a canonical (NVT) ensemble at 250 K. Next, we performed a 1 ns isobaric–isothermal (NPT) simulation at 100 atm and 250 K. We present the VMD snapshots and the MR visualization of the initial hydrate at these pressure and temperature conditions in Figure 1a,b, respectively. This initial hydrate configuration is 2 orders of magnitude larger than published atomistic methane hydrate formation studies.^25,50−54^ Next, we melted the hydrates in the left and right quarters of the simulation box at 400 K while fixing the middle region at 250 K. This resulted in the partially melted system shown in VMD and MR as Figure 1c,1d, respectively. Using the direct coexistence method^55^ as detailed in Adibifard and Olorode,^31^ we found the equilibrium temperature to be 281.5 ± 1.5 K at this pressure of 100 atm. So, we maintained the entire simulation box at 283 K and 100 atm to obtain the equilibrated system shown in Figure 1c,1d and confirmed that the hydrate/fluid interface remained stationary.

It is worth noting that Figure 1c does not clearly show the three gas nanobubbles in the domain because it is a 2D snapshot from the front of the simulation box. In contrast, the MR snapshot in Figure 1d clearly shows three spherical nanobubbles in the simulation box. The first two are on either side of the interface, whereas the third is distributed across the eight vertices of the simulation domain. We would also like to clarify that the screenshots from the MR environment are inadequate at representing the ability to walk around the domain and interact with the interior of the domain in 3D.

### Estimation of Hydrate Growth Rate

We conducted NPT simulations at 250 K and 100 atm to simulate hydrate growth using the system shown in Figure 1c as the starting point. This temperature is much lower than the equilibrium temperature, leading to the movement of the left hydrate/fluid interface further to the left and the movement of the right interface further to the right of the simulation domain because of hydrate formation. Further details on these simulation results are deferred to the next Section. We used the template-matching algorithm^56^ to count the number of hydrate cages in the simulation box at different time steps. Combining this with the known mass of each hydrate cage, we estimated the mass rate at desired output times. As in Adibifard and Olorode,^31^ we created five slices of images in the xy-plane and estimated the number of hydrates in each slice because the hydrate/fluid interface could be nonplanar. The mathematical procedure for determining the instantaneous hydrate growth rate per unit area (*J*_H_) is similar to that presented in Adibifard and Olorode,^31^ but with a positive sign instead of a negative sign in the final expression for *J*_H_
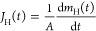
4where *J*_H_ is the instantaneous hydrate growth rate per unit area, *A* is the slice area, *t* is time, and *m*_H_ is the hydrate mass. Section S4 of the Supporting Information provides further details on the derivation of this approach for estimating hydrate growth rate.

## Results and Discussion

### Effect of Gas Nanobubble on Hydrate Growth Rate

**Figure 2:**
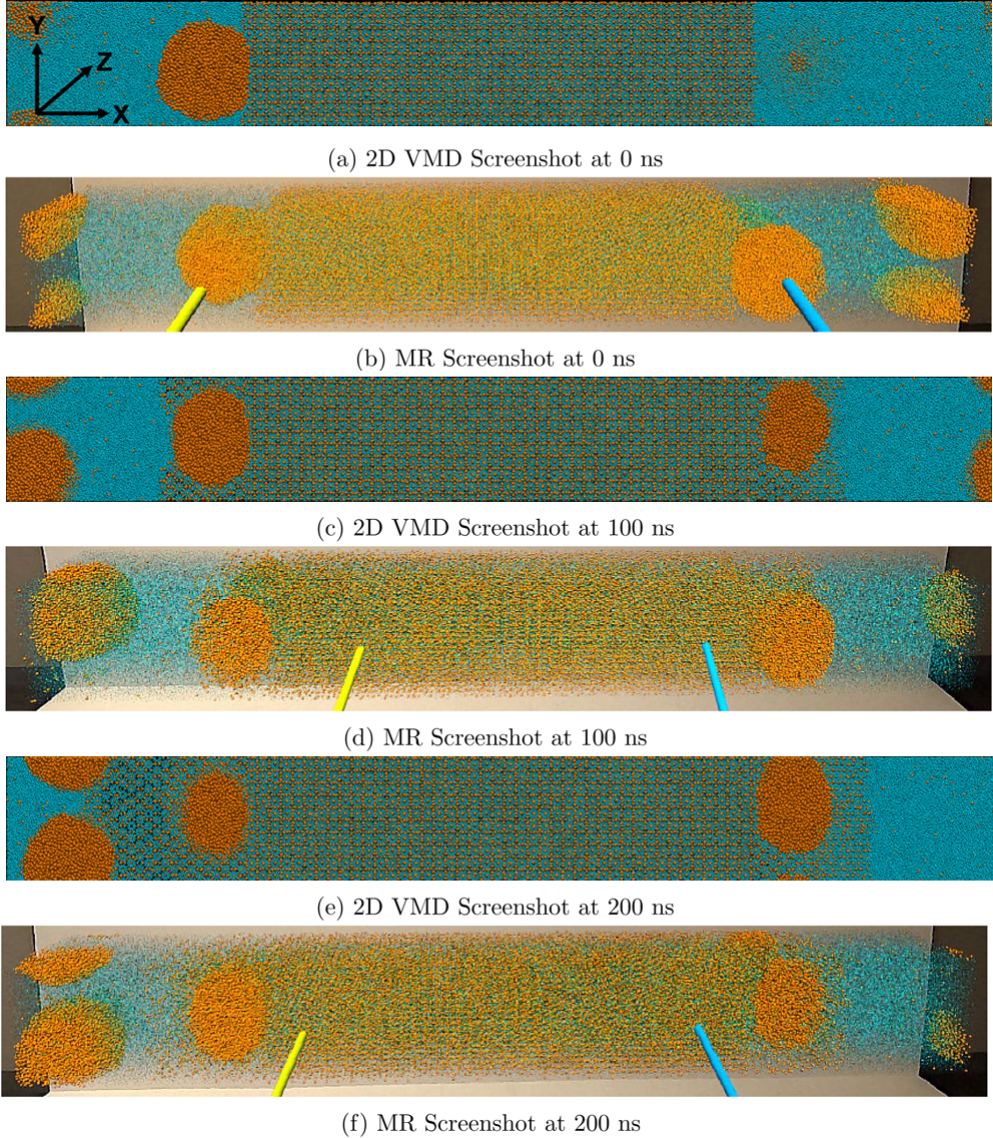
Images show (a) 2D VMD screenshot of the equilibrium structure at 0 ns, (b) MR screenshot at equilibrium conditions, (c) 2D VMD screenshot after 100 ns, (d) MR screenshot after 100 ns, (e) 2D VMD screenshot after 200 ns, and (f) MR screenshot after 200 ns. The orange and cyan spheres represent the methane and water molecules, whereas the yellow and blue sticks are the VR controllers for scaling and rotating the simulation box in MR. The *X*, *Y*, and *Z* axes for all images in this figure are shown in panel (a).

Figure 2 presents 2D VMD snapshots of the front of the simulation box at equilibrium and at specific time steps during the growth of the methane hydrate. It presents the results obtained when the system in Figure 1c is subjected to a lower temperature of 250 K in an NPT ensemble. All the snapshots presented in Figure 2 show the presence of gas nanobubbles on both sides of the simulation box. In Figure 2a, which is identical to Figure 1c because no time steps have been taken at 0 ns, the gas nanobubble on the right side of the simulation box is not clearly visible. This is because it is in the interior of the simulation domain. By rendering the trajectory in the MR environment, we can walk around the nanobubble and rotate it in 3D to obtain clear insights into its geometry and other features. Although Figure 2b more clearly shows the spherical nanobubble, this 2D MR screenshot is inadequate at truly representing the immersive 3D experience of interacting with all parts of the simulation domain. Figure 2c,d show that after 100 ns, the hydrate has grown to trap most of the two gas nanobubbles that were next to the interface at equilibrium. The hydrate growth rate at the hydrate/fluid interface on each side of the simulation box appears similar. However, Figure 2e,2f indicate that the hydrate grew more to the left than to the right of the simulation domain. This observation can be attributed to the presence of a second nanobubble on the left side of the simulation domain, whereas there is no second nanobubble on the right side. This is consistent with the experimentally known idea that gas nanobubbles enhance hydrate growth rate.^57−59^

To confirm the observation that the hydrate growth rate is faster near gas nanobubbles, we simulated a different “replicate” of the initial hydrate configuration in Figure 1a, where a different number referred to as the “initial seed,” is used in a random number generator (RNG). The RNG creates reproducible numbers for sampling initial velocities from the Maxwell–Boltzmann distribution. Using different initial velocities, which are the required starting velocities at the beginning of the simulation, we obtain a different equilibrium hydrate system, shown in Figure S2 of the Supporting Information. The second replicate refers to the group of simulation runs that start from the second equilibrium system initialized with the second initial seed (seed 2). The results from the second replicate in Figure S2 also indicate that the hydrate/fluid interface next to a gas nanobubble moves faster than the interface without a nanobubble in its vicinity. This could be attributed to a mass-transfer limit on the methane gas supply to the growing interface when there is no gas nanobubble close enough to the interface. Between 200 and 400 ns, the gas nanobubble on the left of the simulation domain becomes completely trapped, and the hydrate growth rate slows down. The reduction in the slopes of the curves presented in Figures S4 and S5 and the snapshots of the full domain in Figure S7 of the supplementary file confirm the decrease in hydrate growth rate after 400 ns.

Although this subsection focused on the effect of the gas nanobubbles on the hydrate growth rate, Figure 2 presents the first molecular observation of the trapping of gas nanobubbles within a growing solid hydrate. Considering the novelty of this observation, the next subsection involves detailed visualizations of the process by which the nanobubbles are trapped within the growing hydrate.

### Trapping of Gas Nanobubble within Growing Solid Hydrate

**Figure 3:**
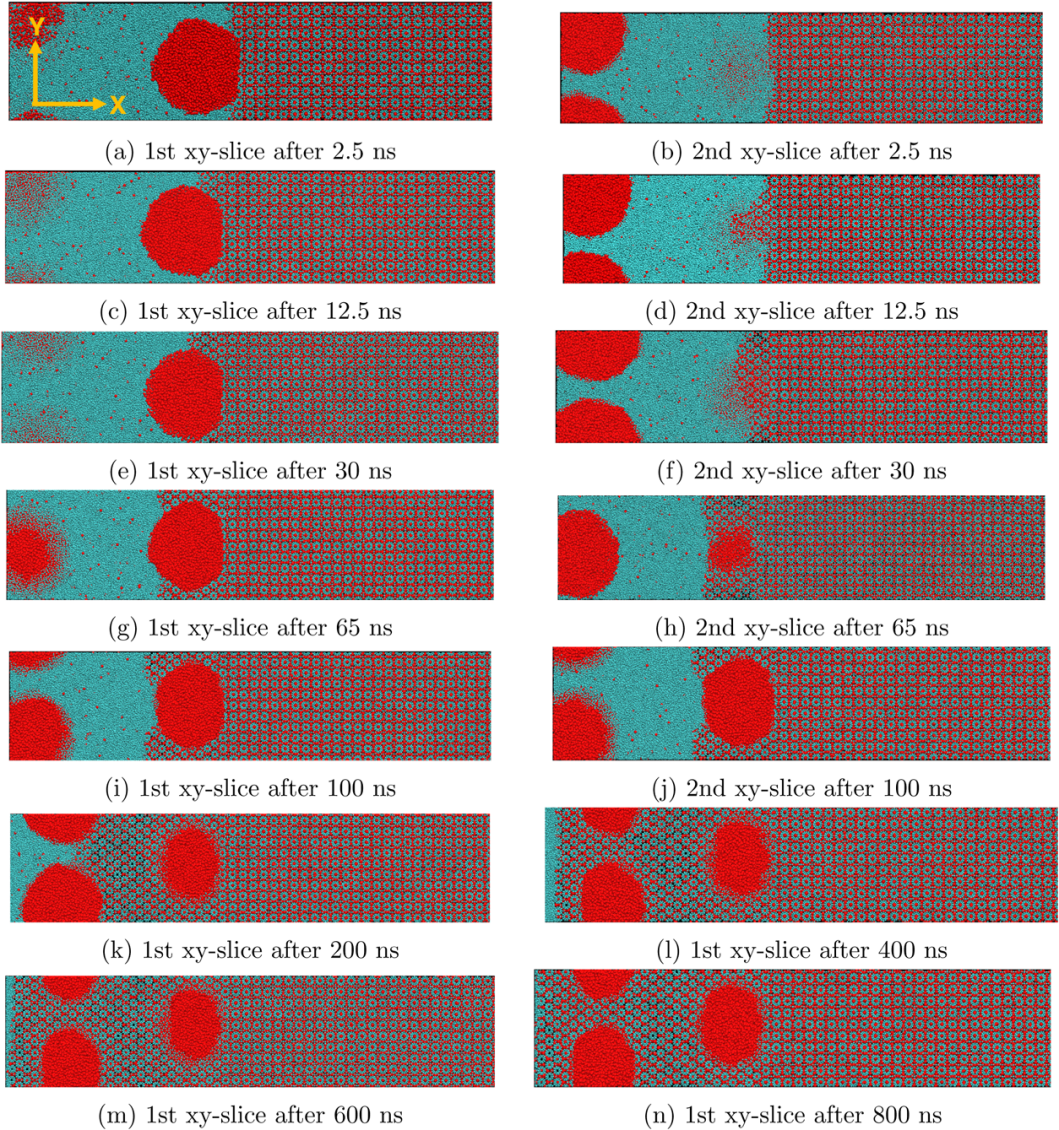
(a) First *x*–*y* slice after 2.5 ns, (b) second slice after 2.5 ns, (c) first slice after 12.5 ns, (d) second slice after 12.5 ns, (e) first slice after 30 ns, (f) second slice after 30 ns, (g) first slice after 65 ns, (h) second slice after 65 ns, (i) first slice after 100 ns, (j) second slice after 100 ns, (k) first slice after 200 ns, (l) first slice after 400 ns, (m) first slice after 600 ns, and (n) first slice after 800 ns of hydrate growth. These results illustrate methane hydrate growth in the presence of methane gas nanobubbles. The snapshots of the first and second slices indicate that the circular gas nanobubbles are spherical, and they get trapped as nanobubbles within the growing solid hydrate.

A closer look at the structure of the hydrates formed over the simulated time interval indicates that the hydrates formed during the MD simulation appear less densely packed than the hydrates initially in the simulation domain. So, we estimated the average methane density of initial and formed hydrates and observed that the average methane density of the hydrates formed is approximately 29% less than that of the initial hydrate. This density difference can be attributed to the incomplete cage occupancy of the newly formed hydrate. To obtain insights into the dynamics of the nanobubbles’ trapping within the growing solid hydrates, we visually inspected two sets of images of *X*–*Y* planes or slices, each set with a different, unique *Z*-value, over the simulated duration, as in Adibifard and Olorode.^31^Figure 3 presents 2D VMD snapshots of the first two slices at different output times. These snapshots focus on the hydrate/fluid interface on the left side of the domain to enable a detailed analysis of the mechanism by which a gas nanobubble is trapped. We also inspected the simulation domain in the MR environment to confirm the observations from the 2D snapshots.

Figure 3a,3b present the results at 2.5 ns when the left hydrate interface is in contact with the nearest methane gas nanobubble. The observed change in the diameter of the nanobubbles from the first slice to the second slice in these two images indicates that the nanobubble geometry is spherical rather than cylindrical. A constant diameter across all *X*–*Y* slices would indicate a cylindrical geometry. Our inspection of the nanobubbles in MR confirmed their spherical geometry. As the simulation evolves, Figure 3c,d show that the hydrate grows outward toward the nanobubble at 12.5 ns. This makes the interface nonplanar, as shown in Figure 3d. The nonplanarity could be attributed to the nonuniform mass transfer or supply of methane gas to the hydrate/fluid interface. After 30 ns, the interface appears planar again, as shown in Figure 3e,3f. At this point, about half of the nanobubble close to the left interface has been trapped within the growing hydrate.

Figure 3g,3h show that the gas nanobubble near the left interface is almost completely trapped within the growing solid hydrate after 65 ns. After 100 ns, the first and second slices in Figure 3i,j indicate that the gas nanobubble is completely trapped within the hydrate. These two images also show that the hydrate/fluid interface becomes concave toward the second gas nanobubble. The curvature can be attributed to a Laplace pressure effect between the gas nanobubble and the surrounding fluid at the interface. After 200 ns, Figure 3k shows that the interface becomes relatively planar again, with about half of the second gas nanobubble trapped within the growing solid hydrate. The second nanobubble is completely trapped at 400 ns, as shown in Figure 3l. Comparing the rate of movement of the hydrate/fluid interface in Figure 3l through n to the corresponding movement of the interface between Figure 3i,k,l, we can infer that the hydrate continues to grow after 400 ns, but at a much slower pace. This qualitative observation is quantified by estimating the hydrate growth rate in the last section of this paper.

A closer inspection of the geometry of the gas nanobubbles before and after trapping (in Figure 3) indicates that the nanobubbles appear spherical before trapping. They become slightly more ellipsoidal in cross-section after they are trapped within the hydrate. This transition from a spherical to an ellipsoidal shape could be attributed to the faster release rate of methane molecules from the left, right, front, and back sides of the gas nanobubble as the hydrate/fluid interface moves past it in the *x*-direction. We computed the density and volume of the gas in the nanobubbles after 0 and 800 ns and observed a 1.53% increase and 47.87% decrease, respectively. The negligible change in density compared to the volume change implies that some of the methane molecules in the nanobubble before trapping got released during the formation of the surrounding hydrates. To obtain clearer insights into the apparent change in nanobubble geometry, we used our MR workflow to visualize the internal structure of the hydrate. This was done at different stages during the trapping of the nanobubble next to the left hydrate/fluid interface. Future studies will focus on understanding the mechanisms responsible for the nanobubbles’ change in shape.

**Figure 4:**
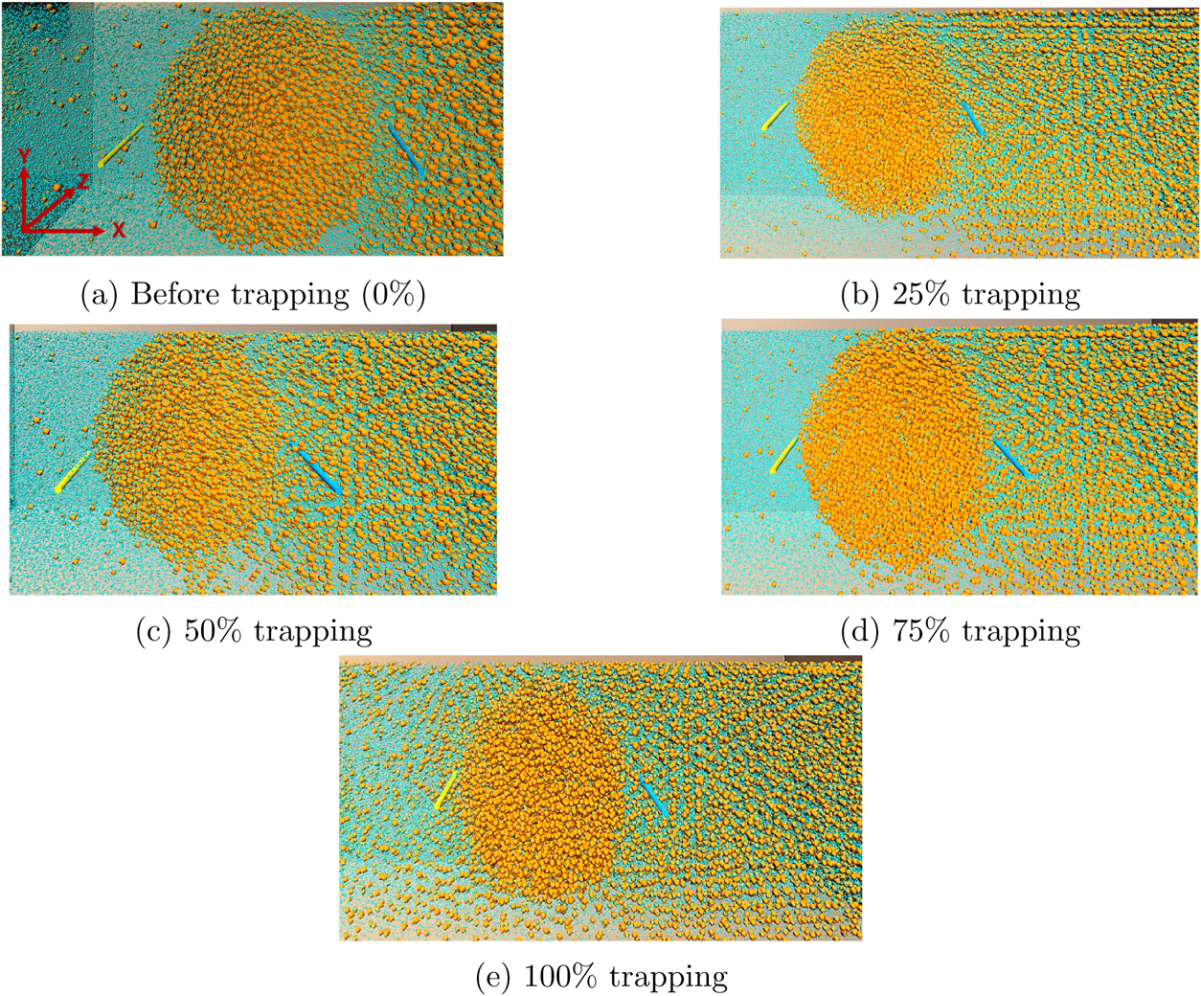
MR snapshots show the gas nanobubble (a) before trapping, (b) after 25% trapping, (c) after 50% trapping, (d) after 75% trapping, and (e) after 100% trapping. The orange and cyan spheres represent the methane and water molecules. In contrast, the yellow and blue sticks are the VR handles for controlling the orientation and scale of the simulation box in the MR environment. The *X*, *Y*, and *Z* axes for all images in this Figure are shown in panel (a). The results show that the spherical nanobubble became ellipsoidal as it got trapped within the growing solid hydrate.

Figure 4 presents the MR snapshots when 0, 25, 50, 75, and 100% of the nanobubble is trapped. The results show that the gas nanobubble, which initially looked spherical before trapping, became progressively more ellipsoid (or “egg-shaped”) as it got trapped within the growing solid hydrate. To the best of the authors’ knowledge, this is the first observation of a spherical-to-ellipsoid transition in shape as a nanobubble gets trapped within a growing solid hydrate. Our novel MR visualization of the internal structure of the growing hydrate in Movie S3 of the Supporting File enabled this observation. Although this section focused on hydrate growth on the left side of the simulation domain, we obtained similar results for hydrate growth past the other nanobubbles in the domain. This is shown in Section S2 of the Supporting Information. The next section presents a detailed procedure to estimate the degree to which the trapping of nanobubbles can enhance the amount of gas stored in hydrates by weight.

### Enhancement of Natural Gas Storage in Hydrates

This subsection presents an approach for estimating how much methane (CH_4_) nanobubbles will boost the average methane storage weight (wt)% and density. Figure S7(c) of the Supporting Information presents the full and annotated version of the results in Figure 3n. It corresponds to the system after 800 ns when the hydrate growth rate is curtailed because of the limited amount of dissolved methane left in the system. The system simulated is 96.24 nm × 12.03 nm × 12.03 nm. So, we estimated the volume of the region containing the new hydrates and trapped nanobubbles (*V*_nH+nb_) as the sum of the volume on the left quarter and half of the right quarter of the domain.

We visualized this system in mixed reality and measured the lengths of the simulation box and the diameter of all three nanobubbles using a physical measuring tape. By scaling the measured lengths (in inches) with the known dimensions of the simulation box (in nm), we estimated the diameters of all three nanobubbles. This yielded average diameters of 8.70, 6.39, and 6.39 nm along the major, intermediate, and minor axes, respectively. So, the sum of the volume of the three ellipsoidal nanobubbles (*V*_nb_) is
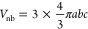
5where *a*, *b*, and *c* are the radii of the major, intermediate, and minor axes, respectively. Excluding *V*_nb_ from *V*_nH+nb_, the volume of only the new hydrates formed (*V*_nH_) is
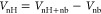
6To estimate the methane density in the hydrate (ρ_ch_), we first estimate the volume of the sI unit hydrate cell (*V*_u_). Using methane’s molecular weight (MW_c_) of 16.032 g/mol, the Avogadro number (*N*_avo_), and considering that the number of methane molecules in the unit cell (*n*_c_) is eight, the methane density in a unit cell (ρ_ch_) is
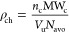
7The same approach in eq 7 was used to estimate the density of water in the hydrate (ρ_w_) as 0.7904 g/cm^3^. The percentage by weight of the methane (CH_4_ wt %) in the region with the newly formed hydrate and trapped nanobubble is computed as follows
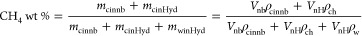
8where *m*_cinnb_, *m*_cinHyd_ and *m*_winHyd_ represent the mass of methane in the nanobubbles, mass of methane in the hydrate, and mass of water in the hydrate, respectively. To compare this storage wt % to the methane weight percent in a pure hydrate with no nanobubble (CH_4_ wt %_ph_), we estimated CH_4_ wt %_ph_ from a ratio of the mass of the eight molecules of CH_4_ in an sI unit cell to the total mass of the eight and 46 molecules of methane and water in the unit cell
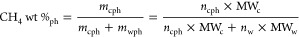
9Here, *m*_cph_, *m*_wph_, *n*_cph_, *n*_wph_, and MW_w_ represent the mass of methane in the pure hydrate, mass of water in the pure hydrate, number of methane molecules in the pure hydrate, number of water molecules in the pure hydrate, and molecular weight of water, respectively. The last paragraph in Section S4 of the Supporting File discusses the methane density in the nanobubbles and in the water solution. Table 1 summarizes the sI-unit cell hydrate parameters^60^ and other parameters used in this Section.

**Table 1:** Outline of Unit Cell Parameters

parameters	magnitudes
ρ_ch_	0.122 g/cm^3^
ρ_w_	0.7904 g/cm^3^
ρ_cinnb_	0.460 g/cm^3^
*n*_c_	8
*n*_cph_	46
MW_c_	16.032 g/mol
MW_w_	18.015 g/mol
*N*_avo_	6.022 × 10^23^
*a*	4.350 nm
*b*	3.195 nm
*c*	3.195 nm

A comparison of the percentage by weight of methane (CH_4_ wt %) in the newly formed hydrate region (18.3%) to that in the pure hydrate (13.4%) shows that nanobubble trapping enhanced methane gas storage by approximately 37%. It is worth clarifying that the molecular simulations that formed the basis of these calculations are applicable to the phenomena occurring at the hydrate/fluid interface in bulk or macroscopic systems. So, the practical application of nanobubble-enhanced gas storage will involve supplying more methane to facilitate further hydrate growth and trapping of more nanobubbles. In contrast, the hydrate growth in the small closed system simulated becomes negligible because of the limited availability of dissolved methane molecules needed to form new hydrates. This is the rationale for ignoring the water-only region on the right of the domain in Figure S7(c) in these calculations.

Further analysis of our simulation results shows that the density of the methane in the methane/water fluid phase decreases with time after the nanobubbles are completely trapped. Figure S6 of the supplementary file plots this density change over time.

### Hydrate Growth Rate

**Figure 5:**
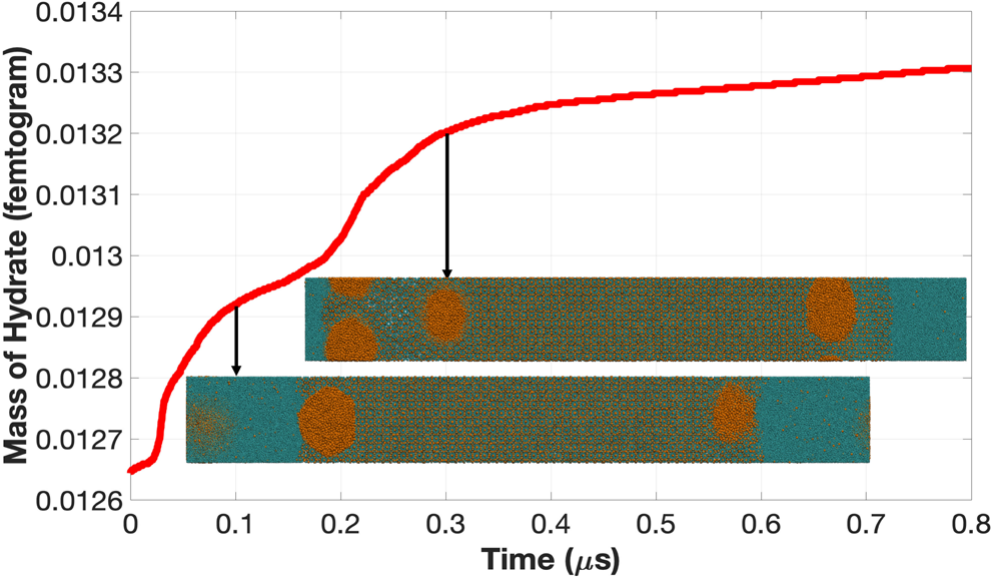
Plot of the evolution of the hydrate mass over time indicates that the hydrate grows faster while the nanobubbles are being trapped. The growth rate decreases significantly after the nanobubbles are trapped.

This subsection presents the results of estimating the hydrate growth rate using the procedure discussed in Section S.4 of the Supporting Information. Figure 5 plots the mass of the hydrate in the simulation box over the 800 ns of NPT simulation performed. The hydrate growth rate is the slope of this plot, which shows considerable changes over the first 400 ns, after which it is approximately constant. Recalling the discussion of Figure 3, the slope changes between 0 and 400 ns occur because the gas nanobubbles accelerate the hydrate growth when the hydrate/fluid interface is in contact with them. This is consistent with experimental observations.^57−59^ The hydrate growth rate is much less after 400 ns. Although there are three gas nanobubbles in the simulation box, only two humps are visible in the plot. This can be attributed to the observation that the trapping of the two nanobubbles closest to the left and right sides of the hydrate occurs simultaneously (between 0 and 100 ns). This explains why the steepest portion of the first hump is steeper than that of the second hump, which occurs during the trapping of the third nanobubble (the farther one from the left interface). The inserted images at 100 and 300 ns confirm that the two close nanobubbles on the two sides of the hydrate are completely trapped at 100 ns, whereas the third nanobubble is completely trapped at 300 ns. It is interesting to observe that the growth rate, as indicated by the slope of the plot, reduced considerably after the first two nanobubbles were trapped. Similarly, after the third nanobubble was trapped, the slope reduced between 300 and 400 ns and remained constant between 400 and 800 ns.

### Conclusions

We performed large-scale molecular studies of methane hydrate growth in systems up to 2 orders of magnitude larger than previous atomistic methane hydrate formation studies. To obtain new insights from this study, we used a combination of mixed reality visualization and multiple 2D VMD snapshots of the front and cross sections of the simulation box. The novel observations from this study are as follows:

1. This work presents the first molecular observation of the trapping of methane gas nanobubbles within a growing solid hydrate.
2. The analysis of the hydrate growth rate and position of the hydrate/fluid interface after trapping the nanobubbles indicates their effect on the hydrate growth rate.
3. Our MR visualization of the internal structure of gas hydrates is the first of its kind. It showed that the spherical geometry of the gas nanobubbles in water gradually became ellipsoid as they got trapped within the growing hydrate.
4. The mixed reality visualization of the internal hydrate structures facilitated the accurate measurement of the gas nanobubbles’ average diameter as 8.8 nm in the fluid solution.
5. This work also shows that gas nanobubble trapping enhanced methane storage in gas hydrates by approximately 37%.

In conclusion, using the mixed reality workflow presented to inspect the internal structure of gas hydrates and other three-dimensional molecular trajectories can facilitate the observation of new insights that could be difficult to observe using standard visualization tools.

## Supporting Information Available

Summary of computational resources; additional figures on two simulated replicates; further details on the approach for computing hydrate mass growth rate; and links to VMD and MR movies of the simulated systems (PDF)
