# Supplementary material for: Molecular Studies and Advanced Visualization of the Trapping of Methane Nanobubbles during Hydrate Growth
Source: J Phys Chem B. 2025 Apr 7;129(15):3860–8. doi: 10.1021/acs.jpcb.4c07851 (PMC12010326; doi:10.1021/acs.jpcb.4c07851)
Supplement: Supplementary file 1 — jp4c07851_si_001.pdf [file jp4c07851_si_001.pdf]

Supplementary Information for: Molecular Studies and Advanced Visualization of the Trapping of Methane Nanobubbles During Hydrate Growth

Temitayo Adeyemi and Olufemi Olorode*

Department of Petroleum Engineering, Louisiana State University, Baton Rouge, Louisiana, 70803, USA.

E-mail: folorode@lsu.edu

This PDF file includes:

Supplementary text

Figures S1 to S7

Movies S1 to S3

Introduction

This supplementary file describes the computational resources used in our large-scale molecular dynamics simulations. It also presents some additional information on the kinetics of gas hydrate formation and our observation of the trapping of methane gas nanobubbles within the growing solid hydrate.

S1 - Computational Resources

The coarse-grained MD simulations conducted are computationally expensive, so we used LAMMPS with the GPU package in all simulations. We used GPU-enabled high-performance computers (HPCs) at the Center for Computation and Technology (CCT) at Louisiana State University (LSU) and obtained a performance of approximately 65 ns/day on four nodes. The specifications of these HPCs are summarized as follows:

1. SuperMike III: This is a 1,285 Tflops HPC with 183 CPU nodes (each node has 64 2.6 GHz cores) and 8 GPU nodes (each has 4 NVIDIA A100 GPUs). The standard nodes have 256 GB RAM, whereas the big memory nodes have 2 TB RAM.

2. QB3: This is an 857 Tflops HPC with 202 CPU nodes (each node has 48 2.4 GHz cores) and 8 GPU nodes (each has 2 NVIDIA Volta V100 GPUs). The standard nodes have 192 GB RAM, whereas the two big memory nodes have 1.5 TB RAM.

S2 - Illustration of hydrate growth

Two different initial seeds were used to generate the initial velocities required to start the two simulation cases presented. The two distinct groups of simulation runs beginning with these initial seeds are called replicates. The initial seed used in the first replicate (presented in the manuscript) is referred to as "seed 1". Similarly, the initial seed used in the second replicate is referred to as "seed 2". Simulating replicates with specified seeds facilitates the reproducibility of the results and allows us to confirm the observations presented in the manuscript. Fig. S1 provides additional information for the replicate discussed in the manuscript. The full system configurations after 2.5 ns, 12.5 ns, 30 ns, and 65 ns are presented here. This contrasts the manuscript, where only a subset of these results were shown for brevity. It is evident from Fig. S1 that hydrate growth and nanobubble trapping are observable at the left and right sides of the simulation domain.

Fig. S2 shows the results obtained for the second replicate. All the phenomena observed

in the first replicate presented in the paper and Fig. S1 are also observed in Fig. S2. However, nanobubble trapping is not observed on the right side of Fig. S2 because we do not have a nanobubble on that side of the simulation domain. Instead, we observe a cylindrical gas accumulation in an apparent gas phase[1]. Furthermore, Figs. S1 and Fig. S2 show that the physical mechanisms discussed in the manuscript occur at about the same time in both replicates. For instance, we observe nonplanar interfaces at 12.5 ns, which become planar again at 30 ns in both replicates. Furthermore, the Laplace pressure effect is evident in both replicates after 100 ns.

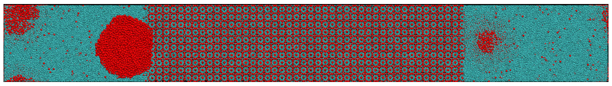

(a) 1st xy-slice after 2.5 ns

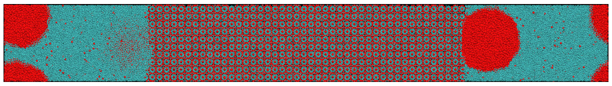

(b) 2nd xy-slice after 2.5 ns

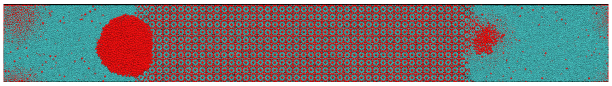

(c) 1st xy-slice after 12.5 ns

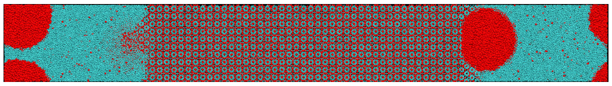

(d) 2nd xy-slice after 12.5 ns

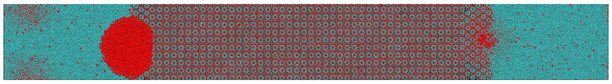

(e) 1st xy-slice after 30 ns

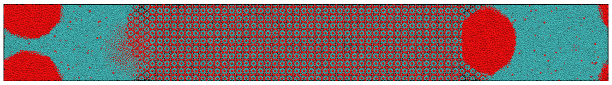

(f) 2nd xy-slice after 30 ns

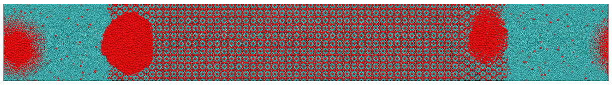

(g) 1st xy-slice after 65 ns

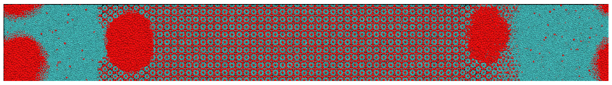

(h) 1st xy-slice after 100 ns

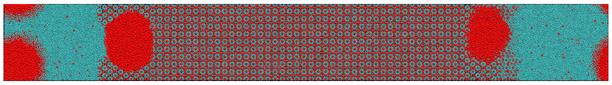

(i) 2nd xy-slice after 100 ns

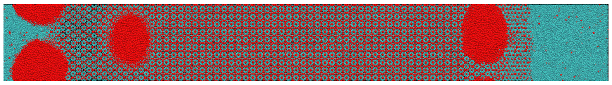

(j) 1st xy-slice after 200 ns

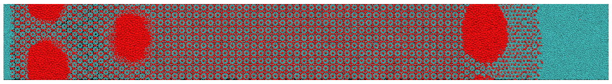

(k) 1st xy-slice after 400 ns

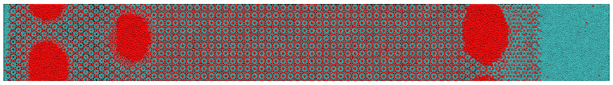

(l) 1st xy-slice after 600 ns

Figure S1: Images show how the hydrate grows over time in the full simulation domain for the first replicate.

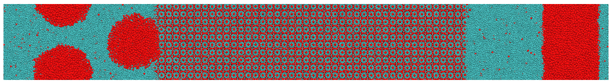

(a) 1st xy-slice after 2.5 ns

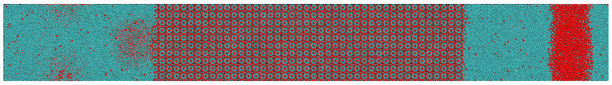

(b) 2nd xy-slice after 2.5 ns

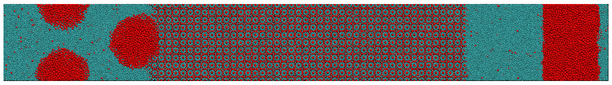

(c) 1st xy-slice after 12.5 ns

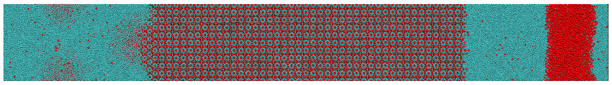

(d) 2nd xy-slice after 12.5 ns

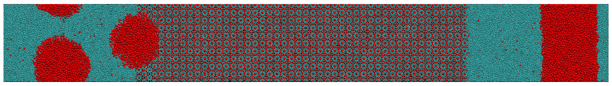

(e) 1st xy-slice after 30 ns

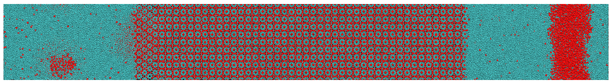

(f) 2nd xy-slice after 30 ns

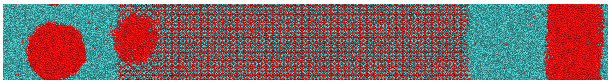

(g) 1st xy-slice after 65 ns

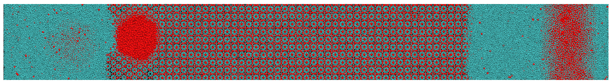

(h) 1st xy-slice after 100 ns

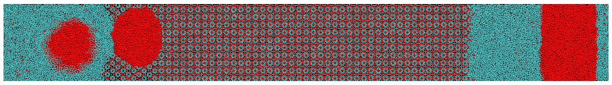

(i) 2nd xy-slice after 100 ns

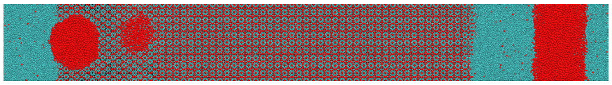

(j) 1st xy-slice after 200 ns

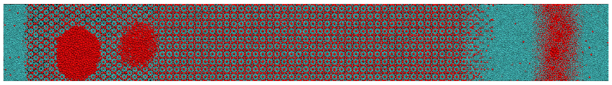

(k) 1st xy-slice after 400 ns

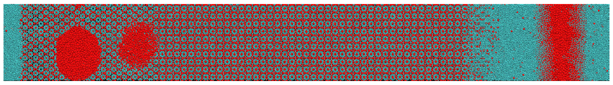

(l) 1st xy-slice after 600 ns

Figure S2: Images show how the hydrate grows over time in the full simulation domain for the second replicate.

S3 - Gas nanobubble trapping in the second replicate

This section presents cross-sections of the simulation results that show the gas hydrate growth in the second replicate. The idea is to validate the observations from the replicate discussed in the manuscript. Physical mechanisms, such as the change in interfacial planarity (Figs. S3d and S3e), nanobubble trapping (Figs. S3d through S3l), and Laplace pressure effect (Fig. S3j), are observed in Fig. S3.

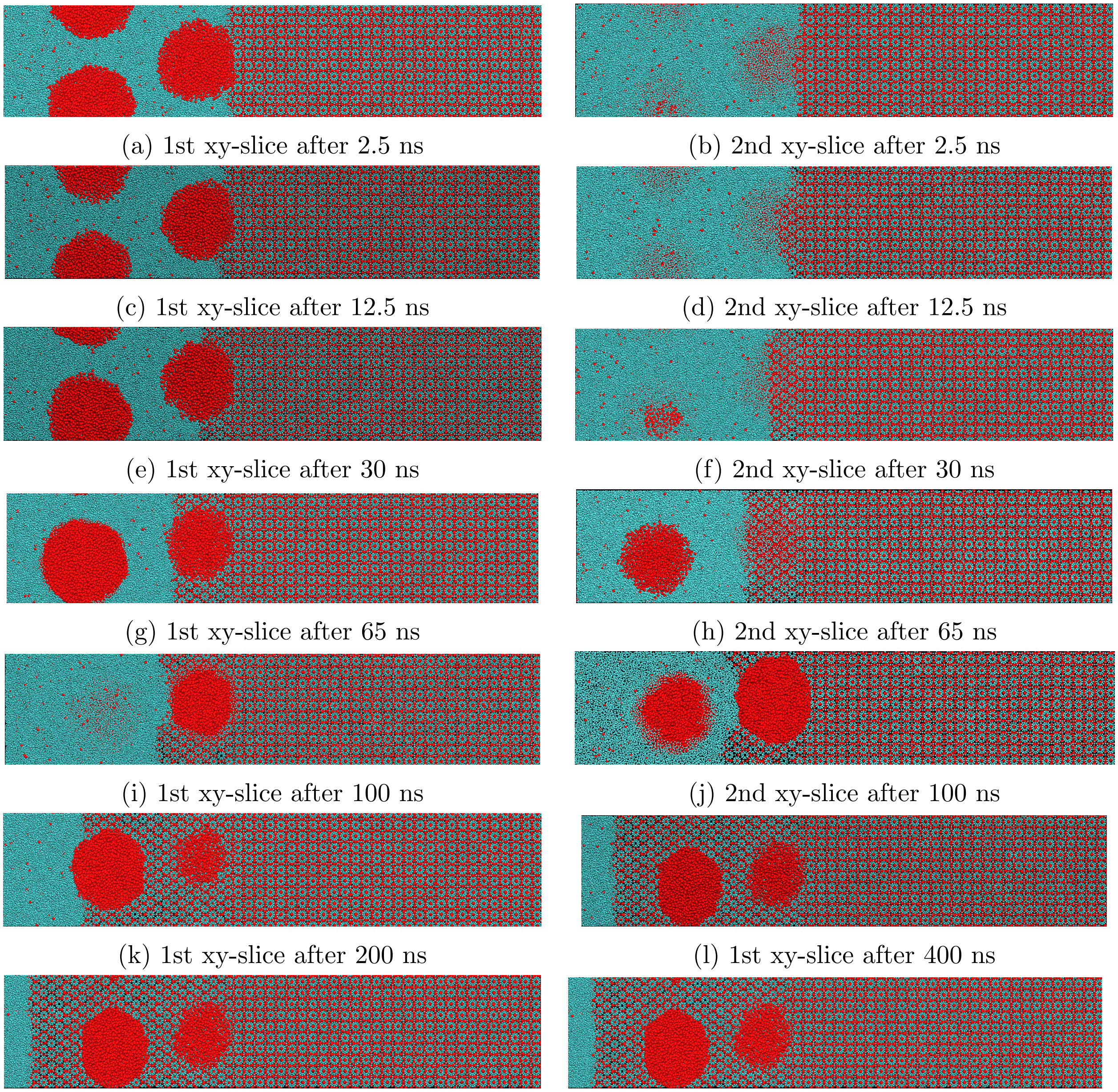

(a) 1st xy-slice after 2.5 ns
(b) 2nd xy-slice after 2.5 ns
(c) 1st xy-slice after 12.5 ns
(d) 2nd xy-slice after 12.5 ns
(e) 1st xy-slice after 30 ns
(f) 2nd xy-slice after 30 ns
(g) 1st xy-slice after 65 ns
(h) 2nd xy-slice after 65 ns
(i) 1st xy-slice after 100 ns
(j) 2nd xy-slice after 100 ns
(k) 1st xy-slice after 200 ns
(l) 1st xy-slice after 400 ns
(m) 1st xy-slice after 600 ns
(n) 1st xy-slice after 800 ns

Figure S3: The images illustrate the hydrate growth of the second replicate in the presence of methane gas nanobubbles. The snapshots of the first and second slices indicate the sphericity of the gas nanobubbles and that they get trapped within the growing solid hydrate.

S4 - Time-dependent evolution of hydrate mass

This section describes the steps involved in estimating the mass of the hydrate in the simulation domain at any specified time step. The hydrate mass in the simulation box at any output time (t) is given as follows:

$$m_H\left(t\right) = N_H\left(t\right) \times m_{HU}, \tag{1}$$

where $m_H\left(t\right)$ represents the hydrate mass, $N_H\left(t\right)$ represents the number of hydrate unit cells, and m_{HU} is the hydrate mass per unit cell, which is estimated as follows:

$$m_{HU} = V_{HU} \times \rho_H, \tag{2}$$

where V_{HU} is the hydrate unit cell volume ($V_{HU} = 1.728\ nm^3$) and ρ_H is the hydrate density (0.9 gcc). The value of $N_H\left(t\right)$ varies over the simulated duration. It is calculated as follows:

$$N_H\left(t\right) = N_z \sum_{i=1}^{N_s} N_{xy,i}\left(t\right), \tag{3}$$

where $N_{xy,i}\left(t\right)$ is the number of hydrate unit cells in the xy-plane of the i-th slice at time t. It is estimated using the template-matching algorithm[2]. The template matching algorithm is a procedure that counts the number of times a small repeating pattern (or template image) occurs in an image. Further details and accompanying code on the implementation of this algorithm can be found in Adibifard and Olorode, 2023[3]. This approach requires the images of the top and bottom halves of a hydrate unit cell as input. It then counts how many times these half-unit cells occur in the image for the entire simulation domain at a specified output time. The term $N_{xy,i}\left(t\right)$ in equation (3) is estimated as follows:

$$N_{xy,i}\left(t\right) = \frac{N_{xy,i}\left(t\right)^{top} + N_{xy,i}\left(t\right)^{bottom}}{2}, \tag{4}$$

where $N_{xy,i}(t)^{top}$ and $N_{xy,i}(t)^{bottom}$ are the numbers of the top and bottom halves of an sI hydrate unit cell. The instantaneous hydrate growth rate per unit area can be calculated as the time-derivative of the mass-time plot, as follows:

$$J_H(t) = \frac{1}{A}\frac{dm_H(t)}{dt}, \tag{5}$$

where $J_H(t)$ is the hydrate growth rate per unit area and A is the area of the hydrate/fluid interface. The average J_H can be estimated by fitting a straight line to the mass-time plot over a specified interval. Fig. S4 compares the rate of hydrate growth for the two replicates. A closer look at Fig. S4 shows that the hydrate growth was faster in the first replicate than in the second replicate. This can be attributed to the presence of more nanobubbles in the first replicate. Fig. S4 also shows that the system approaches a constant and negligible growth rate for both replicates after about 400 ns. As shown in Fig. S5, the hydrate growth was faster on the left side of the simulation box because of the effect of the nanobubble on that side of the domain.

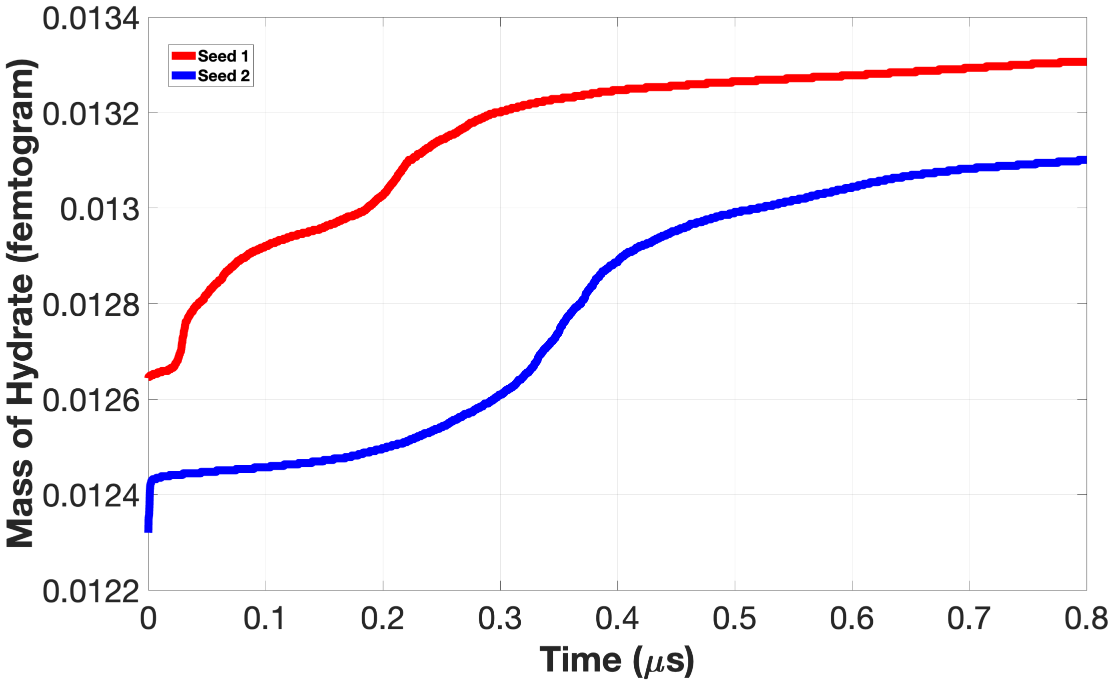

Figure S4: Evolution of the hydrate mass with simulation time for both replicates.

Fig. S5 presents a graphical description of the hydrate growth rate at both sides of the simulation box. It was obtained by dividing the snapshot of the simulation box for each time frame into two halves along the x-axis. The template matching algorithm was then implemented independently on the images for each half of the simulation domain. Fig. S5 confirms that the hydrate grew faster on the left side of the simulation. This can be attributed to the presence of more nanobubbles on that side of the simulation box.

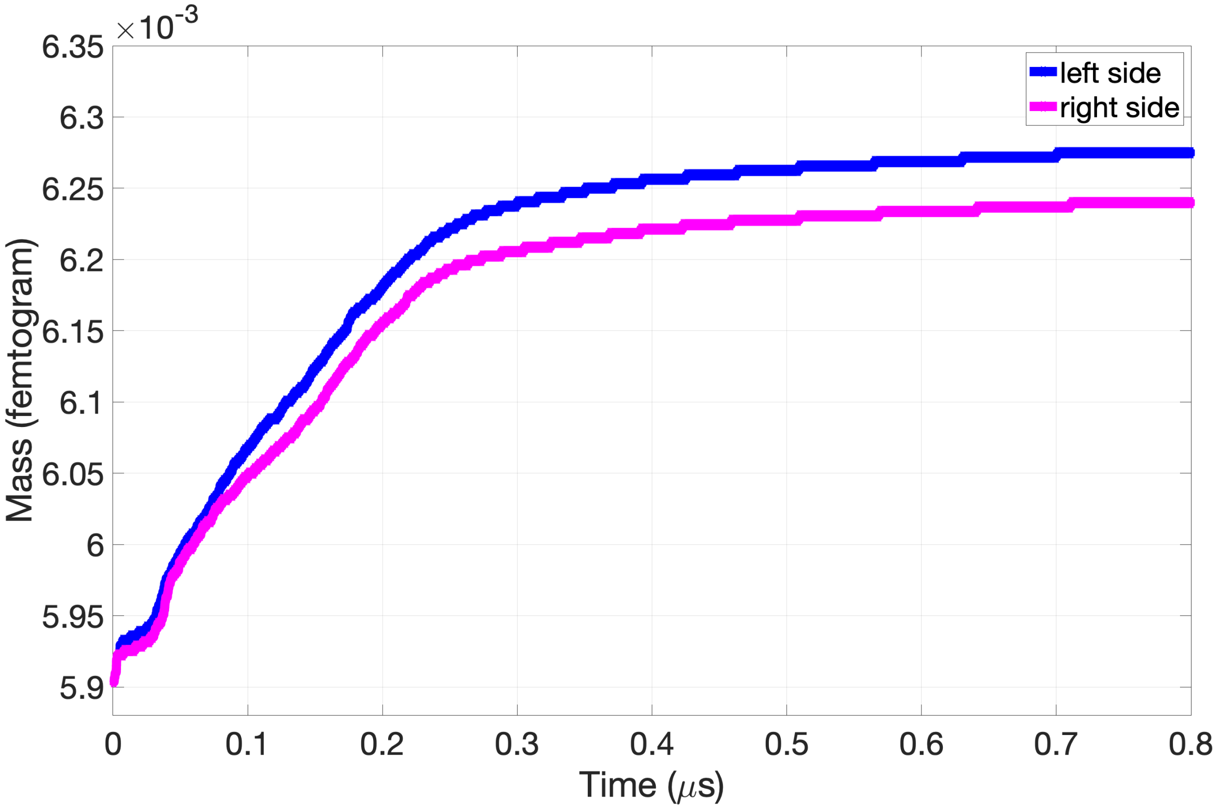

Figure S5: Evolution of the hydrate mass on both sides of the simulation box for the first replicate.

Further analysis indicates that the methane density in the nanobubble is significantly higher than its density in the methane/water fluid phase. Our estimate of the methane density in the methane/water fluid phase at 0 ns was 0.00856 g/cc, and that in the nanobubble was 0.460 g/cc. This implies that the methane density in the nanobubble is approximately 54 times its density in the methane/water fluid phase. These results are consistent with the observation of a significant decline in the hydrate growth rate when there is no gas nanobub-

ble in the fluid phase. The graph below (Fig. S6) also shows that methane density in the fluid phase decreases with time because the methane molecules dissolved in water become trapped within the growing hydrate cages.

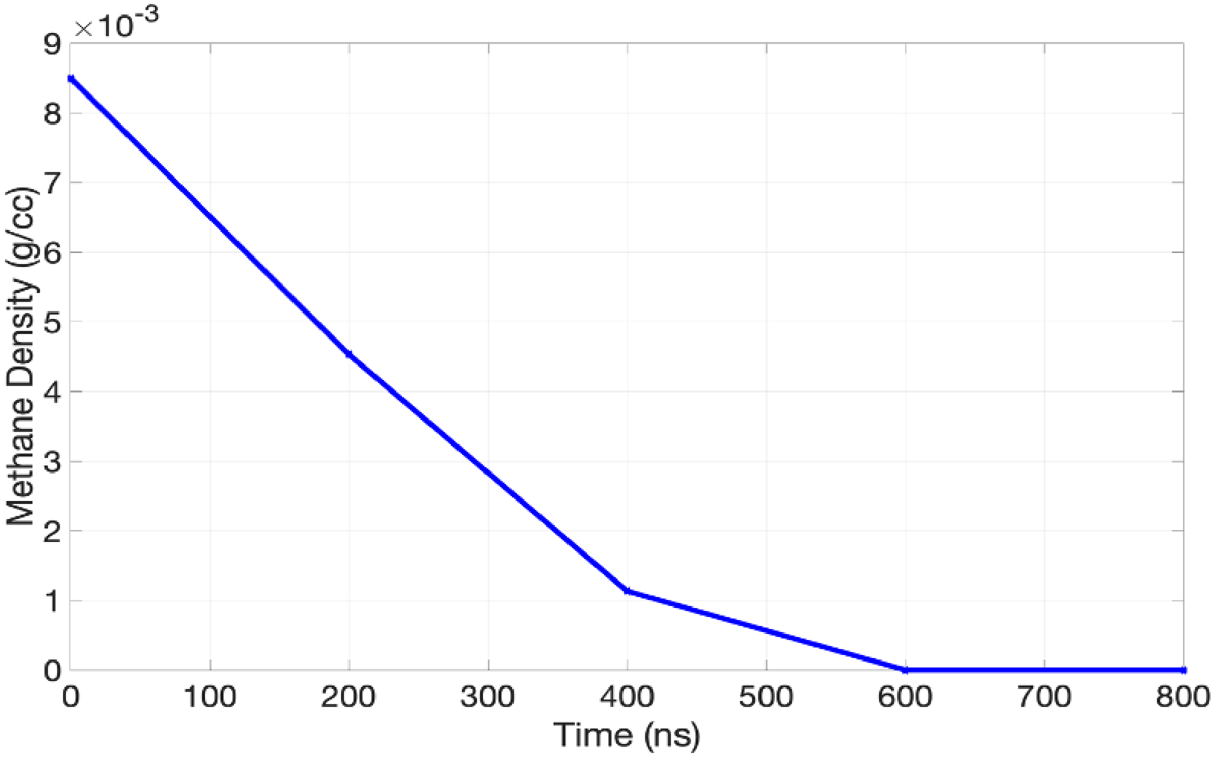

Figure S6: The plot indicates that the density of methane in the fluid phase declines with time as methane gas becomes trapped in hydrate cages.

S5 - Hydrate growth from 400 ns to 800 ns for the first replicate

Fig. S7 presents the first replicate's molecular trajectories between 400 and 800 ns. It confirms the slow hydrate growth indicated by the reduced slope of the red curve in Fig. S4 after 400 ns. This slope decline can be attributed to the absence of methane molecules to facilitate further hydrate growth.

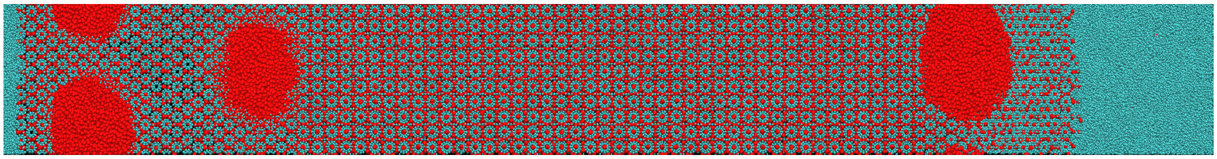

(a) 400 ns

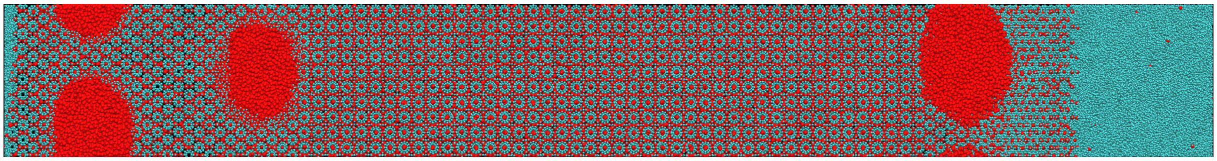

(b) 600 ns

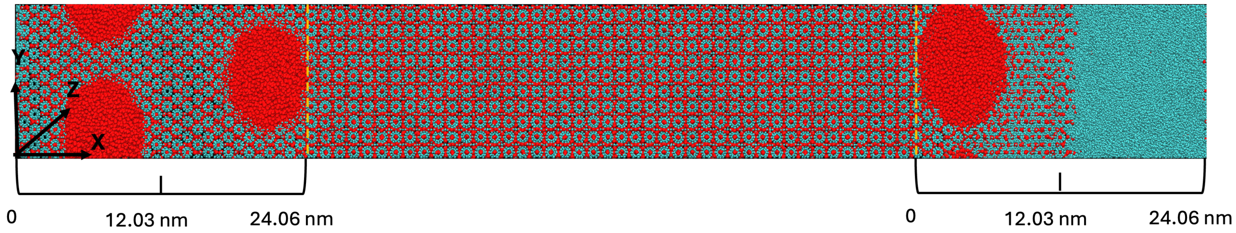

(c) 800 ns

Figure S7: VMD snapshots of the hydrate simulation box after (a) 400 ns, (b) 600 ns, and (c) 800 ns. It shows a decline in the hydrate growth after 400 ns.

Movie S1

The movie provided at `https://youtu.be/Ze7MV3HEpQo` presents the methane hydrate growth over time during the NPT simulation of the case presented in the manuscript. It shows how the hydrate grows to trap the three methane gas nanobubbles that were initially in the fluid phase at equilibrium conditions.

Movie S2

The movie provided at `https://youtu.be/FOy71qQia5Q` presents the methane hydrate growth over time during the NPT simulation of the second replicate. It shows how the hydrate grows to trap the two methane gas nanobubbles that were initially in the fluid phase at equilibrium conditions.

Movie S3

The movie provided at `https://youtube.com/shorts/aXtnRhMsdus` illustrates the size and geometry of the nanobubble as visualized in mixed reality. It shows how we can easily interact with the molecular trajectories to obtain new insights from the simulation results using the mixed reality workflow presented.

References

(1) Bagherzadeh, S. A.; Alavi, S.; Ripmeester, J.; Englezos, P. Formation of methane nanobubbles during hydrate decomposition and their effect on hydrate growth. *The Journal of chemical physics* **2015**, *142*.

(2) Brunelli, R. *Template matching techniques in computer vision: theory and practice*; John Wiley & Sons, 2009.

(3) Adibifard, M.; Olorode, O. Large-Scale Nonequilibrium Molecular Studies of Thermal Hydrate Dissociation. *The Journal of Physical Chemistry B* **2023**, *127*, 6543–6550.
